# GJA1-20k, a Short Isoform of Connexin43, from Its Discovery to Its Potential Implication in Cancer Progression

**DOI:** 10.3390/cells14030180

**Published:** 2025-01-24

**Authors:** Sarah Fournier, Jonathan Clarhaut, Laurent Cronier, Arnaud Monvoisin

**Affiliations:** 1 Laboratory Channels and Connexins in Cancer and Cell Stemness (4CS), UR 22751, University of Poitiers, 1 Rue Georges Bonnet, TSA 51106, CEDEX 09, 86073 Poitiers, France; sarah.fournier@univ-poitiers.fr; 2 Pharmacology of Antimicrobial Agents and Antibioresistance (PHAR2), INSERM U1070, University of Poitiers; 1 Rue Georges Bonnet, TSA 51106, CEDEX 09, 86073 Poitiers, France; jonathan.clarhaut@univ-poitiers.fr; 3 University Hospital Center of Poitiers, 2 Rue de la Milétrie, 86021 Poitiers, France

**Keywords:** GJA1-20k, Cx43, trafficking, mitochondria, cancer progression, alternative translation

## Abstract

The Connexin43 transmembrane protein (Cx43), encoded by the *GJA1* gene, is a member of a multigenic family of proteins that oligomerize to form hemichannels and intercellular channels, allowing gap junctional intercellular communication between adjacent cells or communication between the intracellular and extracellular compartments. Cx43 has long been shown to play a significant but complex role in cancer development, acting as a tumor suppressor and/or tumor promoter. The effects of Cx43 are associated with both channel-dependent and -independent functionalities and differ depending on the expression level, subcellular location and the considered stage of cancer progression. Recently, six isoforms of Cx43 have been described and one of them, called GJA1-20k, has also been found to be expressed in cancer cells. This isoform is generated by alternative translation and corresponds to the end part of the fourth transmembrane domain and the entire carboxyl-terminal (CT) domain. Initial studies in the cardiac model implicated GJA1-20k in the trafficking of full-length Cx43 to the plasma membrane, in cytoskeletal dynamics and in mitochondrial fission and subcellular distribution. As these processes are associated with cancer progression, a potential link between Cx43 functions, mitochondrial activity and GJA1-20k expression can be postulated in this context. This review synthetizes the current knowledge on GJA1-20k and its potential involvement in processes related to epithelial-to-mesenchymal transition (EMT) and the proliferation, dissemination and quiescence of cancer cells. Particular emphasis is placed on the putative roles of GJA1-20k in full-length Cx43 exportation to the plasma membrane, mitochondrial activity and functions originally attributed to the CT domain.

## 1. Connexin43 (Cx43)

### 1.1. Generalities

**Figure 1:**
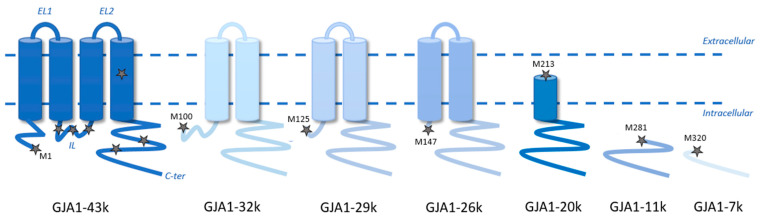
Topology of Connexin43 and of its multiple endogenous isoforms.

Connexin43 (Cx43) belongs to a family of proteins composed of 21 members within the human genome. Among them, Cx43 is the most abundant and ubiquitously expressed [1]. All connexins share a common protein structure with four transmembrane domains and two extracellular and one intracellular loop, with the N- and C-terminal parts on the intracellular side [2] (Figure 1). The N-terminal part is the most conserved among the connexins, while the C-terminal part is the most variable. A connexin is a gap junction protein that forms cell–cell channels, allowing the passage of ions and other small signaling molecules between coupled cells [3]. Six connexin monomers oligomerize to form a transmembrane channel named a connexon or hemichannel. Two connexons of adjacent cells dock together to create an intercellular channel, and clusters of these channels constitute a GAP junction [4]. Surprisingly for a multimeric transmembrane protein, the half-life of Cx43 is relatively short, between 1 and 3 h [5]. Connexin-forming channels are permeable to a wide variety of molecules with a molecular weight up to 1.2 kDa [6], allowing the bidirectional passage of ions (Na^+^, Cl^−^, K^+^), metabolites (glucose), secondary messengers and signaling molecules (IP_3_, calcium, AMPc, ATP and glutamate) [7,8,9,10] and also miRNA, including in a heterocellular context [11,12]. Opened hemichannels are involved in exchanges with the extracellular medium, whereas junctional channels permit direct exchanges between the cytoplasm of contiguous cells. Alterations in connexin levels or mutations can lead to various diseases, such as cancers [13,14], cardiac pathologies [15,16] and diseases linked to development, i.e., oculodentodigital dysplasia, hearing loss and X-Linked Charcot–Marie–Tooth Disease (CMTX) [17,18,19]. In addition to canonical functions in cell-to-cell communication and hemichannel activity, Cx43 may have intracellular and extracellular actions by channel-independent mechanisms [20] linked to the presence of Cx43 in mitochondrial membranes [21] or in extracellular vesicles [22].

Connexin genes share a common structure, with at least two exons separated by one or more introns of different sizes [23]. As is the case with the majority of connexins, the Cx43 gene (*GJA1*) is constituted of two exons. The first exon encodes the 5′ untranslated region (UTR), while the second exon encodes 16 base pairs of the 5′ UTR, the entire coding sequence and the 3′ UTR. The 5’ UTR contains an internal ribosome entry site (IRES), which has the potential to facilitate cap-independent translation [24]. This regulatory mechanism has also been found to control the expression of Cx26 and Cx32 [25,26].

GJA1 mRNA may undergo an alternative translation initiation at in-frame AUG codons to generate six shorter isoforms of Cx43 [27] (Figure 1). These N-terminal truncated proteins are named based on their molecular weight: GJA1-32k, GJA1-29k, GJA1-26k, GJA1-20k, GJA1-11k and GJA1-7k. GJA1-20k, the focus of this review, is synthesized from a methionine at position 213 (M213) and is usually the most abundant isoform found in cells and tissues and consequently the most studied. To date, only one study has examined the function of the other isoforms. When individually transfected into HEK293FT cells, the results showed that GJA1-11k translocated to the nucleus, blocked cell cycle progression and consequently inhibited cell growth [28], while the other isoforms had no significant effects.

Above is a schematic representation of the protein structures of full-length Cx43 (GJA1-43k) and its six different endogenous alternatively translated isoforms (GJA1-32k, GJA1-29k, GJA1-26k, GJA1-20k, GJA1-11k and GJA1-7k). Stars mark the Methionine locations for the alternative translations corresponding to AUG start sites of the different N-terminal truncated Cx43 isoforms, *EL1* and *EL2*, the extracellular loops, *IL,* the intracellular loop and *C-ter,* the carboxyl-terminus.

### 1.2. Cx43 Carboxyl-Terminal Domain Functions

The Cx43 carboxyl-terminal domain (Cx43 CT), which accounts for approximately 40% of the protein from residues 232 to 382, presents a large complexity in terms of interaction and regulation capacities (for review, [29]). Thus, this domain includes multiple phosphorylation sites (serine, threonine and tyrosine) and can regulate Cx43 cellular traffic, gap junction assembly, degradation and channel gating [30,31,32]. Additionally, acetylation, nitrosylation and SUMOylation processes are described to expand the control opportunities of Cx43 turnover, distribution or activity. Cx43 also presents a vast interactome with the CT, displaying numerous protein binding sites that interact with various partners (tubulin, Src, ZO-1 and CCN3/NOV) [29,33]. These protein–protein complexes enable direct interactions with microtubules or indirect interactions with the actin-based cytoskeleton, as well as modulation of a wide range of transduction pathways and channel functionalities [29,34,35,36,37,38,39]. Therefore, through both channel-dependent and -independent mechanisms, Cx43 impacts cellular processes related to tumor progression, such as cell growth, apoptosis and migration. This aspect will be further explored in Section 3.2.3.

## 2. GJA1-20k Isoform

### 2.1. Expression and Regulation of GJA1-20k

GJA1-20k, spanning from amino acid 213 to the final amino acid 382, comprises part of the fourth transmembrane domain and the entire carboxyl-terminal domain of Cx43, so it cannot form a hemichannel but retains all the characteristics of the Cx43 CT domain. As a result, GJA1-20k can be unexpectedly observed when Cx43 expression is studied by Western blotting with antibodies directed against the Cx43 CT domain. The first description of a specific band at 20 kDa was reported in zebrafish heart [40]. In 2007, it was shown that this fragment is not a degradation product of the full-length Cx43 but rather a natural by-product of Cx43 [41]. Finally, in 2013, Smyth and Shaw demonstrated that internal translation of *GJA1* mRNA is responsible for the production of the different Cx43 isoforms, including GJA1-20k [27].

In vitro, GJA1-20k is expressed in several primary cells (endothelial, fibroblasts, keratinocytes, cardiomyocytes, neurons, astrocytes, etc.) as well as in cancer cell lines derived from different types of tumors (breast, lung, brain and prostate). In vivo, GJA1-20k has been identified in multiple tissues, including the heart and the brain, where the majority of studies have been conducted to elucidate its functions. Mice specifically deficient in GJA1-20k, generated by mutating the methionine 213 corresponding to the initiation codon to a leucine, exhibited sudden cardiac death shortly after birth [42]. An exhaustive list of the expression profile and associated functions of GJA1-20k, as described, is summarized in Table 1.

Although GJA1-20k has been implicated in several diseases (see Section 2.2) and found expressed in several cell lines, little is known about the molecular signaling pathways controlling its expression. The generation of GJA1-20k results from alternative translation of *GJA1* mRNA [27,53,54], but the mechanisms regulating this process remain a topic of ongoing discussion. Ul-Hussain et al. first identified a cap-independent process due to the presence of putative IRES activity in the coding region of *GJA1* [54]. Conversely, Salat-Canela et al. demonstrated that the 5′ UTR sequence is necessary for the translation and the generation of GJA1-20k, which occurs via cap-dependent ribosomal scanning or active upstream translation [53]. Lately, Zeitz et al. reported the presence of three different *GJA1* 5′UTR isoforms in human cells due to alternative promoter usage arising from unique transcription start sites (TSSs) [55]. Interestingly, they found that the shorter *GJA1* 5′UTRs promote full-length Cx43 translation over alternative translation of GJA1-20k. These observations collectively indicate the necessity for further investigations aimed at a more comprehensive understanding of the underlying mechanisms involved in *GJA1* translation and GJA1-20k synthesis. Such investigations should include an evaluation of other modes of translation initiation, such as cap-independent translation enhancers (CITEs), which require a free mRNA 5′ end to function.

Concerning the signaling pathways regulating GJA1-20k expression, few studies have been reported. In vitro stimulation of normal mouse mammary epithelial cells with TGF-β results in an epithelial–mesenchymal transition (EMT), characterized by a Smad3- and ERK1/2-dependent decrease in GJA1 20k expression [48,55]. In aging mouse hearts, an increased level of active p38, one of the downstream effectors of the TGF-β non-Smad signaling pathway, has been correlated with a decrease in GJA1-20k expression due to the synthesis of *GJA1* 5′UTR variants [55]. In neonatal mouse cardiomyocytes, the inhibition of the Phosphatidylinositol 3-kinase (PI3K)/protein kinase B (AKT)/mammalian target of rapamycin (mTOR) pathway increased the expression of GJA1-20k [27]. The same result was observed by treating Cx43-overexpressing HeLa with rapamycin, a well-known inhibitor of mTOR [53]. In the same study, chemical inhibition or genetic deletion of MAP kinase-interacting serin/threonine-protein kinase ½ (Mnk1/2), as well as inhibition of the EGFR pathway, also led to increased endogenous GJA1-20k expression in both primary and cancer cells.

### 2.2. GJA1-20k and Physiopathologies

Based on data from numerous in vitro and in vivo studies, GJA1-20k appears to clearly play important roles in various cardiac diseases. Loss of GJA1-20k expression in cardiomyocytes [27,42,43,56,57] disrupted the delivery of Cx43 to the intercalated discs (ICDs), specialized structures that connect adjacent cardiac muscle cells and allow the transmission of cardiac action potentials through gap junctions. Reintroduction of GJA1-20k, or mTOR pathway inhibition known to increase GJA1-20k expression, restored Cx43 localization to the basal membrane [27]. Thus, GJA1-20k is responsible for the forward trafficking of cardiac Cx43 to the ICDs, enabling the transmission of cardiac action potentials. Further, transgenic mice specifically lacking GJA1-20k exhibited abnormal electrocardiograms and died at 2 to 4 weeks of age from sudden cardiac death. These abnormalities were correlated with impaired delivery of Cx43 to the ICDs [42].

GJA1-20k has also been characterized as a stress protein, with increased expression levels during ischemia/reperfusion (I/R) injury in both animal models and human patients suffering from ischemic cardiomyopathy [44]. Studies have revealed a close association between GJA1-20k and mitochondria, which correlated with reduced mitochondrial activities such as respiratory capacity and reactive oxygen species (ROS) production.

In cardiac hypertrophy (CH), whether observed in a spontaneously hypertensive rat model or induced in vitro by angiotensin II (Ang II) on cardiomyocytes, there is a downregulation of GJA1-20k expression mediated by Jak2 signaling, associated with reduced gap junction presence at the ICDs [45]. Moreover, overexpression of GJA1-20k in cardiomyocytes alleviated Ang II-induced hypertrophy by restoring Cx43 trafficking back to the cell–cell border. In addition, cardiac hypertrophy is also associated with diminished mitochondrial biogenesis and reduced maximal respiratory capacity. When overexpressed in cardiomyocytes, GJA1-20k restored the mitochondrial membrane potential and reduced ROS production, exerting its cardioprotective effect in CH. In both patients and a mouse model with arrhythmogenic cardiomyopathy, levels of GJA1-20k are reduced in the heart, leading to a decrease in Cx43 expression at the ICDs and contributing to ventricular arrhythmias. Through AAV-based gene therapy, GJA1-20k expression improved gap junction formation and reduced premature ventricular contraction [57]. In the brain, following traumatic brain injury, elevated expression of GJA1-20k in astrocytes facilitated the recovery and survival of neurons, with its beneficial effects attributed to the regulation of mitochondrial function [49]. Moreover, increased expression of GJA1-20k in astrocyte-derived exosomes led to the inhibition of cell apoptosis and the stimulation of mitochondrial autophagy in lesioned tissues through an unidentified mechanism [58].

### 2.3. Cellular and Molecular Mechanisms Involved

The previous results highlight the fact that GJA1-20k acts as a stress protein in certain pathological situations, offering protection against heart and brain injuries. This protective role is currently attributed to its involvement in (i) rearranging and stabilizing the cytoskeleton, (ii) Cx43 trafficking to the cell membrane and (iii) regulating mitochondrial distribution and function.

#### 2.3.1. Cytoskeleton Dynamics and Cx43 Trafficking

Earlier analyses of the full-length Cx43 sequence have shown that the Cx43 CT domain contains a microtubule-binding domain (MTBD), which allow interactions with alpha- and beta-tubulin, with residues ^264^RV^275^ and ^247^YHAT^250^ being critical for this interaction [59,60]. It is likely that GJA1-20k is also capable of interacting with these two subunits and may control the growth and shortening behavior of microtubules. Indeed, GJA1-20k was shown to colocalize in the cytoplasm and to immunoprecipitate with alpha-tubulin [43,47]. Interestingly, Cx43 is associated with EB1, a marker for rapidly growing microtubule plus ends, which is implicated in gap junction formation through its interaction with dynein/dynactin [61].

GJA1-20k also possesses in its sequence one RPEL motif (RPxxxEL), involved in actin binding, corresponding to the last nine amino acids of the protein, where an Isoleucine substitutes for the final leucine (^374^RPRPDDLEI^382^). Modeling and biochemical experiments provided evidence that this RPEL-like domain can theoretically interact with actin. Complexes with GJA1-20k and actin have indeed been found by immunoprecipitation [43]. However, despite the deletion of the RPEL motif, truncated GJA1-20k still colocalizes with actin, suggesting the existence of another interaction domain that is yet to be determined. When overexpressed, GJA1-20k does not affect the overall quantity of actin protein but leads to an increase in both the number and length of thickened F-actin filaments within cells and contributes to the stabilization of the actin network [43,62]. Moreover, high levels of endogenous or exogenous GJA1-20k induce the formation of actin puncta in the cytoplasm. Collectively, these results identify GJA1-20k as an actin capping protein capable of sequestering G-actin in pools and stabilizing filamentous F-actin fibers [62]. This stabilization contributes to orient microtubules to the cellular junctions, which is a crucial step for proper microtubule-based Cx43 delivery to the membrane. Indeed, as observed during heart and brain injuries, GJA1-20k contributes to Cx43 delivery to cell–cell borders in vitro and in vivo [27,42,43,57] to protect cells and tissues. Mutating the internal AUG to specifically inhibit GJA1-20k expression in vivo resulted in impaired trafficking of Cx43 to the plasma membrane and increased the degradation of poorly trafficked Cx43, leading to sudden death in transgenic mice [42].

More generally, it will be of interest to address whether GJA1-20k also interacts (i) with other cytoskeleton-related proteins like Cx43 through its CT domain, (ii) with known microtubule-associated motor/adaptor proteins that regulate trafficking and delivery within the cells and (iii) with proteins known to mediate coupling between the actin and microtubule cytoskeletons, which is particularly important for the regulation of fundamental biological processes such as cell shape and polarity, division and migration.

#### 2.3.2. Mitochondrial Dynamics, Biogenesis and Metabolic Regulation

Analysis of the subcellular localization in different cell types reveals that GJA1-20k is present within the ER/Golgi apparatus and at the outer mitochondrial membrane [44,45,47], differing from the localization of the full-length Cx43 found in the inner membrane [21]. Mitochondrial morphology and distribution are critical features in various biological processes, such as maintenance of cell shape and polarity, regulation of the cell cycle, tissues morphogenesis and embryogenesis (for review, [63]). During cancer migration, mitochondria redistribute to the leading edge of the cells, providing the energy necessary for cytoskeleton rearrangement and consequently cell invasion [64]. In this context of mitochondrial dynamics, GJA1-20k acts as an organelle chaperone, facilitating the distribution of mitochondria to the cell periphery through a microtubule-dependent mechanism. On the other hand, GJA1-20k may associate with mitochondrial membrane proteins, similar to full-length Cx43, which has been found to interact with several mitochondrial partners such as Tom20 [21].

GJA1-20k plays a protective role in various stress conditions across different cell types and organs. In HeLa cells overexpressing GJA1-20k, H_2_O_2_-induced oxidative stress was mitigated by maintaining the mitochondrial network distribution and preventing its fragmentation [47]. In mouse hearts, AAV9-mediated delivery of GJA1-20k, while promoting mitochondrial biogenesis, reduced the mitochondrial membrane potential, respiration and ROS production in order to preserve cardiac function after ischemic injury [44]. In a rat model of cardiac hypertrophy, in vitro overexpression of GJA1-20k in cardiomyocytes enhanced energy metabolism by increasing the mitochondrial membrane potential and respiration and decreasing ROS generation. The reasons for these contrasting effects in different heart injury models remain to be elucidated. Similarly, in a rat model of brain injury GJA1-20k overexpression in astrocytes protected neurons from apoptosis in part by promoting the biogenesis of mitochondria [49].

GJA1-20k overexpression in HEK293 cells led to significant changes in mitochondrial dynamics and structure. It induced the formation of smaller mitochondria due to actin recruitment and the formation of actin filament rings around the mitochondria, which ultimately induced mitochondrial fission, independently of the canonical DRP1-dependent pathway [46]. As a result, there was a decrease in ROS production generated by oxidative stress. During this process of fission, GJA1-20k may act directly as a nucleation factor, since GJA1-20k has been identified as a direct actin-binding protein capable of recruiting and stabilizing actin filaments [62].

GJA1-20k has also been identified as a key mediator of mitochondrial transfer through tunneling nanotubes (TNTs) from astrocytes to neurons [49] and from mesenchymal stromal cells to chondrocytes [65], offering protective effects after traumatic brain injury and osteoarthritis, respectively. GJA1-20k may act on this transfer by regulating the extension of microtubules and actin into TNTs, binding mitochondria with microtubules and bringing Cx43 to the membrane. This is consistent with previous findings indicating that Cx43 plays a role in TNT formation [66].

#### 2.3.3. Other Role(s)

Despite the absence of a nuclear localization signal (NLS), GJA1-20k has surprisingly been found in the nucleus of neural crest cells. In this unexpected location, GJA1-20k formed a complex with the transcription factor BTF3 and RNA polymerase II, which then regulated the transcriptional activity of N-cadherin, a protein known to promote neural crest migration [67]. Interestingly, when Cx43 CT was expressed in cardiomyocytes and cancer cells, it localized to the nucleus and induced a decrease in proliferation. This nuclear signal may be attributed to the presence of GJA1-20k or other Cx43 isoforms, potentially generated by alternative translation of the mRNA produced by the transfected cDNA encoding the Cx43 CT domain [68]. Mennecier et al. identified a 20 kDa immunoreactive protein in the nucleus, detected by Cx43-specific antibodies, which correlated with reduced growth rates in glioma cells [69].

## 3. GJA1-20k and Progression of Cancer

### 3.1. Demonstrated Roles of Alternative Translations During Epithelial-to-Mesenchymal Transition

Unlike in prokaryotes, the process of non-canonical translation in eukaryotes is relatively rare and restricted to specific genes [70]. Among these identified polycistronic genes, several are implicated in cancer progression, apoptosis and metastatic initiation processes. Thus, the *p73* gene, a member of the p53 tumor suppressor family, can produce N-terminally truncated isoforms named DNp73 able to support an EMT-like phenotype switch, with an increased cell motility linked to decreased E-cadherin expression [71]. In melanoma cells, DNp73 interferes with p73, enhancing aggressiveness without affecting cell growth. In addition, two melanoma antigens (MELOE-1 and MELOE-2) derived from a polycistronic RNA were overexpressed in the melanocytic lineage, suggesting a differential translation in normal versus tumor cells [72]. The tissue-specific epigenetic control leading to the specific expression of MELOE-1 and MELOE-2 in melanoma cells suggests that these antigens could be relevant candidates for immunotherapy.

The PI3K/AKT/mTOR signaling pathway directly related to the cell growth, proliferation and apoptosis of prostate cancer cells could also be modulated by cap-independent translation of the mTOR transcript [73]. This regulatory mechanism steers to negative influences during the development and progression of solid tumors. The mTOR signaling pathway not only affects EMT, but also regulates the alternative translation of *GJA1* mRNA, suggesting a transcriptional control of EMT progression. The link between reduced GJA1-20k expression levels relative to Cx43 and the EMT process was demonstrated by James et al. (2018) in a mouse mammary gland epithelial cell line (NmuMG cells) during TGFβ1-induced EMT [48]. GJA1-20k expression was altered by TGFβ1 exposure together with a reduction in junctional Cx43, despite an increase in Cx43 protein stabilization. The effect on Cx43 trafficking, previously observed in cardiac cells [27,43], appears to occur in epithelial cells during the initial stages of induced oncogenic transformation. In the EMT context, the specific effect of TGFβ1 on GJA1-20k was associated with smad3-ERK1/2 activities. Surprisingly, ectopic expression of GJA1-20k in NmuMG cells did not prevent EMT, even when Cx43 trafficking to the membrane and gap junction formation increased. These data suggest that membranous Cx43 does not impact the mesenchymal transition of mammary epithelial cells under TGF β1 stimulation.

### 3.2. Potential GJA1-20k Impacts on Human Cancer Cells

In the 1960s, cancer was the first pathological consequence associated with gap junctional deficiency [74]. Much evidence for a role of connexins has since been provided, demonstrating that connexins can modulate all stages of tumorigenesis through both channel-dependent and -independent mechanisms, from cancer cell growth and dynamics to metastatic dissemination and response to therapies [75,76,77,78]. The roles of connexins in the development of cancer are complex, acting as tumor suppressors or promoters according to their isotype, abundance and localization and to cancer stage. In primary cancer sites, reduced connexin expression or a cytoplasmic misdistribution is frequently observed, even if differences exist according to the connexin and cancer types considered. Notably, most of studies revealed that Cx43 mainly acts as a tumor suppressor in breast, lung, melanoma and prostate cancer [75,79,80,81]. However, Cx43 can also exert a prometastatic role during cancer progression, especially in prostate and breast cancers and glioblastoma [82,83,84,85]. Given the ambiguous role of Cx43 in the cancer phenotype, the level of GJA1-20k, which modifies the proportion of membranous Cx43, must be considered for its impact on the intrinsic properties of cancer cells. This includes potential effects on metabolic and dynamic capacities, as well as on heterocellular interactions during dissemination or within the niches of secondary sites. Along the same lines, given the role played by Cx43 CT in cancer cell abilities and the shared sequence with GJA1-20k, a putative role of the short isoform must be addressed [86,87].

#### 3.2.1. Control of Cx43 Exportation to Plasma Membrane

The increased presence of full-length Cx43 in cancer cells at the plasma membrane may impact their aggressive phenotype at the unicellular level or increase their homocellular or heterocellular gap junctional intercellular communication (GJIC).

Glioblastoma (GBM), one of the most aggressive and lethal brain cancers, is characterized by its ability to diffusely invade surrounding normal brain tissue. As with other cancer types, Cx43 exhibits complex roles in the establishment, progression and persistence of malignant glioma, demonstrating both tumor-promoting and tumor-suppressing functions. This duality can be attributed to the cellular heterogeneity observed in GBM, suggesting that Cx43 may have different roles depending on the cellular population and the state under consideration [88]. The in vitro and ex vivo invasion ability of individual GBM cells was correlated with Cx43 expression level, as demonstrated in the human cell line U251 [89]. In these cells, using an shRNA approach, Cx43 was implicated in the formation and function of invadopodia, mainly by modulating the kinetics of invadopodia formation. This dynamic effect appeared to depend on Cx43 hemichannel activity [90]. Recent findings also demonstrate that connexins play an important role in the microenvironment of malignant glioma. Cx43 intercellular channels can form gap junctions at the edge between GBM cells and astrocytes, facilitating tumor invasion [86,91,92]. Thus, elimination of Cx43 in astrocytes reduced glioma invasion in a murine model, confirming a role in driving tumor invasion [86]. Cx43 expression also enables glioma cells to interact not only with astrocytes but also with other cell types in the brain parenchyma, especially endothelial cells (ECs) [93]. Cx43 expressed in both ECs and GBM cells facilitates direct cell-to-cell communication, which appeared crucial for tube formation [93] and may significantly influence glioma invasion [94]. Functional GJIC between these cells was demonstrated to allow the transfer of a pro-invasive miR (miR-5096) from GBM cells to microvascular ECs. This direct transfer of miR induced Cx43 expression in ECs and promoted EC tubulogenesis [12]. Physical contact and GJIC between astrocytes and GBM cells may also confer chemotherapeutic resistance on GBM cells to temozolomide (TMZ) [95,96]. As endogenous GJA1-20k is expressed in primary astrocytes, ECs and some cancer cell lines (see Table 1), a potentially decisive role of this short isoform can be considered during GBM progression.

Although disagreements persist regarding the prometastatic role of Cx43 during breast cancer progression, a subtype-dependent role for this connexin during propagation and late states was clearly described (for review, [75,97]). As mentioned for GBM, controversies have mainly arisen due to the cellular heterogeneity observed at different stages of dissemination. The essential role for Cx43 appears to occur during dissemination to secondary sites and mainly corresponds to the establishment of heterocellular GJIC, particularly with ECs during the intra- and extravasation processes and with host cells at secondary sites. Thus, Cx43 allowed the formation of functional gap junctions with endothelial cells, enhancing cancer cells transendothelial migration in vitro [98]. Cx43 overexpression increased breast tumor cells diapedesis, whereas blockade of GJIC by carbenoxolone altered the passage throughout the endothelial monolayer. In breast-derived brain metastases, Cx43 expression was upregulated in lesion regions compared to adjacent normal tissue. RNAi depletion of Cx43 or pharmacological blocking of GJIC with carbenoxolone inhibited brain colonization by blocking tumor cell extravasation and blood vessel co-option. The breast cancer metastatic gene *Twist* induced an increase in Cx43 expression and GJIC, leading to amplified extravasation and brain microtumor formation [99]. In vivo, a role for membranous Cx43 was also demonstrated in the vascular adhesion and survival of dormant breast cancer cells in brain metastases [99]. Accordingly, the upregulation of Cx43 in breast cancer micrometastases appears to facilitate their attachment to pulmonary endothelium in a nude mouse model [100].

As reviewed elsewhere [83,84,101], previous works have also linked the metastatic dissemination of prostate cancer (PCa) cells to Cx43 expression levels. In vivo, Cx43 presents modified expression patterns according to PCa progression. In early stages (grade 2), Cx43 expression is strongly decreased within the primary tumor, while re-expression is observed in more advanced stages in the prostate (grade 3) and in bone metastases [52,81]. At the transcript level, dataset analyses revealed that Cx43 exhibits a specific signature in bone secondary tumors, not observed for the two other GJ genes expressed in normal prostatic tissue (corresponding to Cx32 and Cx26) and for the other secondary sites. In vitro and preclinical experiments clearly demonstrated pro-aggressive effects of Cx43 during dissemination steps [52,82,84,102]. Thus, Cx43 overexpression in a low-metastatic model (LNCaP) revealed that Cx43 expression level was related to the aggressiveness of PCa cells, with potentiation of migration ability and increased sensitivity to osteoblastic-conditioned medium (ObCM), used to mimic the metastatic bone niche. In addition to the modulation of Cx43 expression level, altered trafficking to the plasma membrane was also demonstrated in androgen-insensitive PCa cells, especially in cells extracted from bone metastases such as PC3 and C4-2b human cells [103]. In these trafficking-deficient models, ObCM stimulation remained ineffective even in Cx43-overexpressing PCa cells indicating that the presence of Cx43 at the plasma membrane level is required for bone microenvironment sensitivity [52]. It is noteworthy that GJA1-20k is endogenously expressed in LNCaP cells, a PCa cell line characterized by the presence of Cx43 mainly at the plasma membrane. Furthermore, endogenous GJA1-20k expression levels increased in the presence of ObCM [52] or rapamycin in the LNCaP cell model [104]. These results, obtained in a human model without Cx43 exportation defect to the plasma membrane, suggest a potential role of this N-truncated Cx43 isoform in the aggressiveness of PCa cells in the bone context. Preliminary data from our laboratory indicate that GJA1-20k overexpression significantly enhances the exportation of Cx43 to the plasma membrane in a PCa cell model exhibiting a deficiency in full-length Cx43 exportation. Taken together, as initially demonstrated in the cardiac model, these findings unveil a chaperone effect of GJA1-20k that should be considered, given the promigratory role of membranous Cx43 observed during the progression of cancer. Thus, as already demonstrated [82,105], the adhesion capacity of PCa cells to osteoblasts increases in the presence of membranous Cx43 and this effect could be enhanced by GJA1-20k overexpression, particularly in PCa cells subjected to stress conditions. When the full-length Cx43 is exported to the plasma membrane, the Cx43-mediated cell–cell communication may also potentiate the Ca^2+^ flow from osteogenic cells to cancer cells, thereby increasing metastatic ability [87]. Considering the link between mTOR and the regulation of GJA1-20k expression, it is noteworthy that the same group demonstrated that both cell types can form heterotypic adherens junctions (N-cadherin/E-cadherin), leading to mTOR signaling activation and the promotion of bone metastasis progression [106]. Regarding the well-known Cx43 positive effect on cellular adhesion [82,107], GJA1-20k could also impact heterocellular adhesion between tumor and bone cells, leading to optimized bone homing of disseminated cells. Moreover, it was recently demonstrated that circulating tumor cell clusters exhibited heightened metastatic potential compared to isolated cells [108]. These data suggest that increased GJA1-20k may also be involved in protecting these aggregated cancer cells from the shear stress of the bloodstream or from the unfavorable environment found at secondary sites.

#### 3.2.2. Role of Carboxyl-Terminal Domain in Cancer and Analogies for GJA1-20k

GJA1-20k is characterized by the presence of the complete carboxyl-terminal domain of Cx43 in its sequence, which harbors multiple interaction sites with signaling proteins and the cytoskeleton. This structural similarity raises important questions about the functional parallels between GJA1-20k and the biological roles attributed to Cx43 CT (for review, [29]). We can therefore postulate that findings from studies focusing on the role of Cx43 CT in cancer progression might also be relevant to GJA1-20k, particularly in cellular processes such as proliferation, apoptosis or migration.

For instance, Cx43 CT expression in neuroblastoma cells suppressed cell growth by potentially delaying exit from the G1/G0 phase of the cell cycle [109] and, in glioblastoma cells, Cx43 CT reduced anchorage-independent growth [110]. Similarly, in osteosarcoma, Cx43 CT inhibited cell proliferation by downregulation of S phase kinase-associated protein 2 (skp2) expression, an important component of the Skp1-Cullin-F-box protein (SCF) ubiquitin–ligase complex implicated in p27 ubiquitin–proteasome degradation [111]. In cervical cancer, Dang et al. also observed a significant decrease in cell proliferation correlated with the localization of Cx43 CT to the nucleus, where it may influence the expression of cell cycle-related genes [68]. Interestingly, Cx43 CT in breast cancer cells resulted in an increased expression of p53, possibly through the inhibition of miR-125b expression [112].

Overexpression of Cx43 CT in pancreatic cancer cells enhanced apoptosis through direct interaction with the pro-apoptotic protein Bax, thereby initiating the mitochondrial apoptotic cascade [113]. Conversely, in human glioma cells, Cx43 CT was implicated in conferring resistance to the chemotherapeutic agent Temozolomide. Cells expressing a Cx43 CT exhibited decreased resistance compared to those expressing the full-length Cx43 protein [114].

Bates and colleagues [115] demonstrated that expression of CT-truncated Cx43 in rat glioma cells led to a reduced migratory capacity, while the overexpression of Cx43 CT in human glioblastoma cells led to enhanced migration through actin cytoskeleton reorganization [110]. In cervical cancer, cells exhibited also enhanced migration, via p38 activation, when expressing Cx43 CT [116]. In prostate cancer, we demonstrated that Cx43 CT was sufficient to induce the migration of cancer cells stimulated by osteoblast-conditioned medium, correlating with increased active Rac1 [52]. Finally, Cx43 CT has also been implicated in neuronal migration and in B-lymphocytes spreading via Rap1 activation, with both of these processes known to be linked to cancer progression [117,118].

Cx43 CT has emerged as a promising target for the development of mimetic peptides aimed at modulating connexin function in various pathological conditions, particularly cancer. The first peptide described in this context was alpha carboxy terminus 1 (alphaCT1), a 25 amino-acid peptide that mimics the last nine amino acids of the Cx43 CT, which contains the PDZ-binding motif that interacts with zonula occludens-1 (ZO-1) and an antennapedia internalization sequence to facilitate cellular uptake. AlphaCT1 showed anti-cancer effects on glioblastoma, melanoma and breast cancer cells by enhancing chemosensitization and inhibiting tumor growth [119,120,121,122]. Another noteworthy peptide is TAT-Cx43_266-283_, mimicking the Src SH3 binding domain, which has also demonstrated an anti-tumor effect on glioblastoma both in vitro and in vivo by reducing the growth, invasion and progression of malignant gliomas while enhancing survival in glioma-bearing mice [123,124]. Interestingly, TAT-Cx43_266-283_ treatment on GSCs was also associated with a reduced mitochondrial metabolism and distribution [125]. A third peptide, named juxtamembrane 2 (JM2), has been shown to inhibit proliferation and induce apoptosis of melanoma and ovarian cancer cells in vitro by arresting the cell cycle in S phase. In vivo, JM2 has been demonstrated to effectively inhibit both tumor growth and recurrence [126]. Collectively, these connexin-mimetic peptides may influence GJA1-20k functions based on their structural characteristics. It would then be beneficial to re-evaluate their effects, including on mitochondrial function, cellular stress responses and intercellular communication. This reassessment could provide new insights into the mechanisms of action of these peptides and potentially lead to more targeted therapeutic approaches.

#### 3.2.3. Control of Mitochondrial Localization and Activity

A growing body of evidence indicates that tumor development is not solely dependent on the presence of oncogenes or tumor suppressor mutations. Instead, metabolic adaptations play a crucial role in regulating the growth, survival and metastatic potential of tumor cells. Although the precise mechanisms are not fully elucidated, the Warburg effect, described in the 1930s, is a common feature of metabolic reprogramming during cancer progression. This effect leads to a predominant less efficient process of “aerobic glycolysis” than oxidative phosphorylation in mitochondria [127]. However, recent studies have revealed that functional mitochondria are nevertheless essential for tumor cells, even if most of cancer cells present an altered mitochondrial metabolism with an excessive lactate production in the presence of abundant oxygen [128]. The dysfunction of mitochondrial activities can be attributed to a modified stromal–epithelial metabolic coupling, also known as the “reverse Warburg effect”, or to various factors, including mutations in nuclear- or mitochondrial-encoded genes, alterations in the respiratory complexes or mitochondrial products, such as reactive oxygen species (ROS). These imbalances can affect tumor cell growth, apoptosis, EMT, aggressiveness and metastatic competence [129,130,131]. Oxidative phosphorylation, once considered as a “tumor suppressor”, should now be viewed as a potential contributor to tumor cell adaptation in stressful microenvironments. Moreover, alterations in mitochondrial dynamics or plasticity, including biogenesis and mitophagic, fusion or fission processes, are also considerably involved in prostate cancer progression [132].

Given the significant mitochondrial consequences induced by GJA1-20k in cardiac and brain models, along with its interaction with the outer mitochondrial membrane and its role in fission [46], it can be hypothesized that this short isoform also contributes to the regulation of mitochondrial number, size and subcellular localization, contributing to the adaptation and plasticity of tumor cells. Indeed, a clear link between mitochondrial localization and cell migration has been established, demonstrating a redistribution of mitochondria towards the leading edge of cancer cells during migration and an enrichment of mitochondrial proteins in invadopodia during invasion [131,133]. Furthermore, the current consensus suggests that mitochondrial fragmentation ultimately enhances cell motility, invasion and metastasis [134]. Thus, increased mitochondrial fission induced by hypoxia in breast cancer cells has been shown to enhance their invasive ability [135]. Similarly, elevated levels of Mitochondrial Fission Factor (MFF) have been observed in metastatic PCa cells and correlated with poor patients prognosis [136]. Therefore, it would be of interest to analyze the expression level of GJA1-20k in tumors in order to determine whether it rises similarly to other proteins involved in fission such as DRP1, MFF, FIS1 or MiD49. In addition, mTOR, a factor implicated in GJA1-20k expression in cardiac and cancer cells, was also associated with the trafficking of mitochondria to the cortical cytoskeleton, thereby supporting lamellipodia dynamics in epithelial cancer cells [137]. Mitochondrial trafficking is also clearly associated with cancer cell plasticity. Given that GJA1-20k is considered a stress protein during ischemia–reperfusion in myocytes due to its protective role in mitochondrial fission [46,138], we can speculate that GJA1-20k plays a role in the processes of the migration, invasion and adaptation of prostate cancer cells within the metastatic microenvironment.

In the metabolic context, the stromal compartment of carcinomas is of prior importance and largely determines the initiation and promotion of tumors. Among the key players within this microenvironment, cancer-associated fibroblasts (CAFs) are capable of establishing a reciprocal metabolic crosstalk with cancer cells, thereby facilitating the reprogramming of the cancer cell phenotype. Evidence for direct intercellular mitochondrial transfer has already been demonstrated through the formation of open-ended TNTs between adjacent cells [139,140], including interactions between CAFs and prostate cancer cells, which leads to modified OXPHOS activity [141]. Additionally, TNT formation in PCa cells may influence their resistance to therapy [142]. As Cx43 contributes to mitochondrial transfer via regulating TNT formation between mesenchymal and epithelial cells [66,143] and GJA1-20k promotes Cx43 delivery to the plasma membrane, a putative role of GJA1-20k in the potential control of mitochondrial activity by TNTs could also be postulated within PCa. Interestingly, in the context of osteoarthritis, a role of GJA1-20k was recently demonstrated in mitochondrial transfer between mesenchymal stem cells (MSCs) and chondrocytes. GJA1-20k’s impact on MSC mitochondrial transport requires direct cell-to-cell contact and is dependent on the expression levels of Cx43 [65]. These results highlight the interest in investigating the role of GJA1-20k in the potential transfer of mitochondria between MSCs or osteoprogenitors and cancer cells during bone metastases.

Additionally, the involvement of extracellular vesicles (EVs) and of direct Cx43-GJIC in the mechanism for mitochondrial transfer were also shown to retore mitochondrial functional parameters in deficient cells [144]. The upregulation of GJA1-20k in astrocytes was found to be correlated with an increased presence of this short isoform in exosomes, which was associated with enhanced mitochondrial activity, a reduced apoptotic rate and diminished damages to adjacent neurons [50,58,145].

These findings suggest that such an effect could be considered in intercellular communication between cancer cells and other cells within the tumor microenvironment. For instance, many prostate cancer recurrences following curative treatment are thought to arise from the reactivation of disseminated tumor cells (DTCs) and the bone marrow is considered to be an important site for dormant tumor cells, due to the high incidence of bone metastases. These dormant tumor cells are characterized, among other features, by a reversible non-proliferative state (G0/G1 arrest), allowing them to remain viable and represent a potential source for late recurrence and therapy resistance. As previously mentioned, conditions within the osteogenic niche, mainly hypoxia and/or interaction with the tumor microenvironment, may favor the preferential expression of the stress protein GJA1-20k. Consequently, GJA1-20k may exert a metabolic impact during cellular stresses in the bone context and could modulate both the quiescence and metabolic activity of PCa DTCs. Indeed, increased mitochondrial fission and/or resistance to ROS production induced by GJA1-20k are linked with decreased metabolic activity and cellular quiescence [46].

## 4. Concluding Remarks and Perspectives

Among the six isoforms of Cx43, GJA1-20k has been identified as a key player in heart physiology and pathology. As a chaperone protein and regulator of cytoskeleton stability, GJA1-20k promotes GJIC by controlling full-length Cx43 trafficking to the plasma membrane, particularly during cellular stress. This regulatory effect has also been documented in other tissues, such as the brain, and in different cell types, suggesting a conserved mechanism for Cx43 transport. The cellular effects of GJA1-20k observed in the cardiac and nervous systems, together with the established role of Cx43 in tumorigenesis, suggest that the presence of GJA1-20k in cancer cells may be an important new regulatory factor during cancer cell migration and invasion. Further investigations are required to elucidate the specific molecular mechanisms by which GJA1-20k regulates Cx43 trafficking. This includes investigating potential interactions with cytoskeleton-related proteins, microtubule-associated motor/adaptor proteins and proteins mediating actin–microtubule coupling that are critical for cell dynamics. In addition, it will be of interest to determine whether this regulatory mechanism is exclusive to Cx43 or whether it also influences the trafficking of other membrane proteins.

Moreover, increased expression of GJA1-20k in cancer cells may increase Cx43 localization at cell boundaries and improve GJIC. This could potentially amplify the bystander effect during targeted chemotherapy, a hypothesis that merits further investigation due to its implications for cancer treatment strategies. Notably, the delivery of GJA1-20k via adeno-associated virus has shown promise in improving gap junction formation in cardiac models, suggesting that similar strategies could be explored within the cancer context.

GJA1-20k has also been shown to modulate mitochondrial activity and dynamics under stressful conditions, which is essential for cell survival. Although GJA1-20k is expressed in several cancer cell lines, its specific functions remain largely unknown at this point. Cancer cells are frequently exposed to exogenous stresses such as hypoxia and consequently develop adaptive mechanisms, including mitochondrial retrograde signaling or intercellular mitochondrial transfer through TNTs with stromal cells. Several studies have demonstrated that increased mitochondrial fission promotes cancer progression, particularly cell migration and invasion. In this context, mitochondria are preferentially redistributed to the leading edge of the cancer cells. Additionally, a decrease in mitochondrial mass contributes to the survival and dormancy of cancer stem cells. Overall, given the role of GJA1-20k in inducing mitochondrial fission and redistribution, GJA1-20k may play an important role in tumor plasticity, cancer cell metastasis, survival and quiescence in secondary sites such as hypoxic osteogenic bone. In the latter case, quiescent disseminated tumor cells represent a risk to patients but also offer opportunities for early detection. However, it should be emphasized that antibodies specific for the detection of GJA1-20k will also react with full-length Cx43, making it difficult to differentiate between the two in patient biopsies. This also implies that conclusions drawn about full-length-Cx43 using antibodies against the C-terminal domain should be re-evaluated. Furthermore, studies focusing on the proper functions of Cx43 CT should also be reconsidered due to its strict structural homology with GJA1-20k.

In less than a decade, significant advances in our understanding of the role of GJA1-20k in cardiac physiology have provided important new insights into Cx43 trafficking and the regulation of mitochondrial activity. Future studies will aim to determine the potential involvement of this multifaceted isoform of Cx43 in tumor progression, with the intention of providing advances in the field of cancer.

## Figures and Tables

**Table 1:** Expression profile and related functions of GJA1-20k.

Tissue Type	Model	Expression and/or Functions	Ref.
Rat kidney	NRK cell line	First biochemical identification of a 20 kDa natural by-product resulting from Cx43 processing. This short protein is absent in cells after Cx43-specific RNAi treatment or those derived from Cx43-deficient mice and not prevented by the use of proteases inhibitors.	[41]
Murine oral epithelium	AT84 cell line		
Brain endothelium	bEnd3 cell line		
Human heart	Cardiomyocytes	GJA1-20k, the predominant form in the heart, acts as a trafficking chaperone for full-length Cx43 and its expression is under mTOR inhibition control.	[27]
Mouse heart	C57BL/6 mice	GJA1-20k stabilizes actin filaments, guides microtubule machinery, increases Cx43 hemichannel transfer to cardiac intercalated discs and permits the maintenance of Cx43-dependent gap junctional coupling after acute ischemia.	[43]
Mouse heart	C57BL/6 mice	GJA1-20k is an endogenous stress response protein upregulated by ischemic or ischemia/reperfusion conditions. GJA1-20k is present in the outer mitochondrial membrane and promotes mitochondrial biogenesis and metabolic quiescence, leading to cardioprotection.	[44]
Mouse heart	Transgenic C57BL/6 mice (GJA^M213L^)	GJA1-20k protects Cx43 from degradation and maintains normal electrophysiological function in the heart.	[42]
Rat heart	SHR and Wistar-Kyoto rats	Level of GJA1-20k regulates gap junction formation and mitochondrial function to attenuate Ang II-induced pathological cardiac hypertrophy.	[45]
Mouse heart	C57BL/6 mice	GJA1-20k induces focal constriction of mitochondria by actin polymerization, leading to protective mitochondrial fission.	[46]
Human cervix	HeLa cell line	GJA1-20k presents a microtubule-binding domain, mediates mitochondrial transport and maintains mitochondrial network integrity during cellular stress.	[47]
Human skin	HaCaT cell line		
Human kidney	HEK293T cell line		
Mouse brain	Primary fibroblastic and glial cells		
Murine mammary gland	NMuMG cell line	Smad3- and ERK-dependent pathways reduce GJA1-20k expression and gap junction formation. GJA1-20k overexpression is not sufficient to halt epithelio-mesenchymal transition of immortalized cells. GJA1-20k regulates Cx43 oligomerization.	[48]
Rat brain	Neurons and primary astrocytes	Overexpression of GJA1-20K in astrocytes promotes viability and recovery of neurons after brain injury.	[49]
Rat brain	Primary fetal neurons and astrocytes	GJA1-20k uptake, via astrocytic exosomes by damaged neurons, attenuates traumatic brain injury in a rat model.	[50]
Mouse brain	CCM3+/− mouse brain and endothelial cells	GJA1-20k, through interaction with ZO-1, contributes to increased permeability of the blood–brain barrier in cerebral cavernous malformation by promoting structural changes in endothelial cell junctions that favor gap junction formation over tight junction stability.	[51]
Cancer (cell lines) Human prostate	LNCaP cells	In Cx43-overexpressing LNCaP metastatic cells, GJA1-20k expression increases upon bone-conditioned medium stimulation.	[52]
Human lung	A549 and HOP-62 cells	GJA1-20k is detected in various cancer cell lines. Its expression is regulated by Mnk1/2 pathway.	[53]
Human breast	MDA-MB-231 and BT-549 cells		
Human cervical/endometrial	C33A/Ishikawa cells		
Mouse embryonic carcinoma	NF-1		

## Data Availability

Not applicable.
